# Understanding schizophrenia as a self-disturbance

**DOI:** 10.1192/j.eurpsy.2021.2075

**Published:** 2021-08-13

**Authors:** K. Fredheim, R. Wynn

**Affiliations:** 1 Division Of Mental Health And Substance Use, University Hospital of North Norway, Tromsø, Norway; 2 Department Of Clinical Medicine, UiT The Arctic University of Norway, Tromsø, Norway

**Keywords:** Psychosis, psychotherapy, phenomenology, self-disturbance

## Abstract

**Introduction:**

Phenomenolgical theory has contributed to a renewed understanding of schizophrenia, as a supplemental perspective to contemporary operationalistic theories. Phenomenological research with patients suffering from schizophrenia emphasizes the nature of subjectivity and suggests that a basic disturbance of the self can be understood as a core phenotypic marker of shizophrenia.

**Objectives:**

To briefly present and discuss the phenomenological theory of self-disturbance, illustrated through a case.

**Methods:**

In this case study we briefly present the phenomenological theory for self-disturbance. We illustrate the theory by presenting elements from a case involving a patient that suffers from schizophrenia. Our focus in on how the self-disturbance is experienced by the patient and how the therapist can address this experience. The challenges in psychotherapy related to the phenomenon of self-disturbance and the implications for examination and treatment are discussed.

**Results:**

The patient gives a detailed description of how he experiences a diminished basic sense of self. Central elements in his experience are a loss of a common-sense ability, hyperreflexivity, and a loss of a first-person perspective. He describes how this disorder creates difficulties in communication, relationships, treatment, and in coping with life. He also describes which elements of his treatment that he has experienced as the most helpful. The case underlines the importance of considering the concept of self-disturbance in psychotherapy.

**Conclusions:**

In this case study, we draw on phenomenological theory to gain insight into a patient’s experiences relating to the concept of self-disturbance.

**Disclosure:**

No significant relationships.

